# The impact of COVID‐19 on residents of long‐term care facilities with learning disabilities and/or autism

**DOI:** 10.1111/irv.13139

**Published:** 2023-04-26

**Authors:** Elise Tessier, Harriet Webster, Nurin Abdul Aziz, Joe Flannagan, Asad Zaidi, Andre Charlett, Gavin Dabrera, Theresa Lamagni

**Affiliations:** ^1^ COVID‐19 Vaccine and Epidemiology Division UK Health Security Agency London UK; ^2^ All Hazards and Intelligence Division UK Health Security Agency London UK; ^3^ Statistics, Modelling and Economic Division UK Health Security Agency London UK

**Keywords:** autism, care home, learning disabilities, SARS‐CoV‐2

## Abstract

**Background:**

The COVID‐19 pandemic has had disproportionate impact on vulnerable populations including those with learning disabilities. Assessing the incidence and risk of death in such settings can improve the prevention of COVID‐19. We describe individuals who tested positive for SARS‐CoV‐2 while residing in care homes for learning disabilities and/or autism and investigate the risk of death compared with individuals living in their own homes.

**Methods:**

Surveillance records for COVID‐19 infections in England from 02 February 2020 to 31 March 2022 were extracted. Data on property type, variant wave, vaccination, hospitalisation and death were derived through data linkage and enrichment. Care home residents with learning disabilities and/or autism and diagnosed with COVID‐19 were identified and analysed, and logistic regression analyses compared the risk of death of individuals living in private residence. We assessed interaction parameters by post‐estimation analyses.

**Results:**

A total of 3501 individuals were identified as diagnosed with SARS‐CoV‐2 whilst living in 632 care home properties for learning disabilities and/or autism. Of the 3686 episodes of infection, 80.4% were part of an outbreak. The crude case fatality rate was 2.6% and 0.6% among care home residents with autism and/or learning disabilities and their counterparts in households, respectively.

The post‐estimation analyses found over eight times the odds of death among care home residents in 60 years old compared with their counterparts living in private homes.

**Conclusions:**

Care home residents with learning disabilities and/or autism have a greater risk of death from COVID‐19. Optimising guidance to meet their needs is of great importance.

## 1 INTRODUCTION

The COVID‐19 pandemic has had disproportionate impact on certain populations, such as older people and people with underlying health conditions. Residents in care homes are likely to have risk factors such as being of older age or having comorbidities, which make them vulnerable to severe COVID‐19 outcomes. Additionally, these settings are high risk for COVID‐19 transmission due to large household sizes and close contact with multiple carers. As such, attention has been focused on reducing the risk of transmission in the adult social care sector. However, much of the guidance has been geared towards care homes offering long‐term care to older people,^1^ and large national research studies in care homes often exclude residents under 65 years.^2^, ^3^

Adults and children residing in care homes specialising in learning disabilities and/or autism experience intersections of risk; some may have underlying risk factors for severe COVID‐19 outcomes, while living in a care home setting where transmission of COVID‐19 is more likely. Existing societal inequalities can be amplified during outbreaks and pandemics, leading to increased vulnerability to infectious diseases. Previous studies highlight how COVID‐19 impacts people with learning disabilities and/or autism accessing healthcare and public health information.^4^, ^5^

The risk of serious infection and death from COVID‐19 is likely to vary among people with learning disabilities, given the varied nature of these conditions and level of care required. A study conducted by Perera et al. on COVID‐19 deaths among people with intellectual disabilities in the United Kingdom and Ireland concluded that there is a need to stratify guidance based on the risk of severe outcomes so that appropriate levels of shielding can be applied.^6^ Previous research using population level information has also called for further explorations of the risks of COVID‐19 for those with learning disabilities and/or autism living in residential care homes.^7^ Elucidating the risk of serious infection and death in long‐term care facility settings could therefore improve the prevention of COVID‐19 outcomes for this population.

In this paper, we identify and describe individuals who tested positive for SARS‐CoV‐2 while residing in long‐term care facilities specialised in learning disabilities and/or autism. We investigated the risk of death among individuals in these settings compared with individuals living in their own homes.

## 2 METHODS

### 2.1 COVID‐19 cases

The UK Health Security Agency (UKHSA) is responsible for collecting data on all notifications of COVID‐19 infection in England via the Second Generation Surveillance System (SGSS).^8^ We extracted data for positive COVID‐19 cases (polymerase chain reaction or lateral flow test) from 02 February 2020 to 31 March 2022, when free community testing was discontinued.^9^ For individuals with more than one COVID‐19 episode, each diagnosis occurring ≥90 days from the last episode was counted as a new episode. For each individual, data on age, sex, ethnicity, index of multiple deprivation (IMD) deciles, address and region were collected from the first episode extracted from SGSS.

### 2.2 Residential setting of cases

When booking tests or reporting COVID‐19 results, individuals are asked for the residential address of the person being tested. We enhanced residential addresses of all COVID‐19 episodes through geospatial address matching to obtain a Unique Property Reference Number (UPRN) and a Basic Land Property Unity (BLPU) class, which indicates the property type based on local authority permitted use.

A BLPU class of RI01, a Care Quality Commission (CQC) ID indicating a care home, and/or addresses with a mention of ‘care home’ were used to identify individuals residing in care homes at the time of infection diagnosis. Individuals where the reinfection was not at the same property type were excluded from the analyses.

To determine which cases were living in long‐term care facilities for learning disabilities and/or autism, we identified residential care settings (excluding shared living and positive lives settings) from the CQC list of providers where the sole service users were people with learning disabilities and/or autism. CQC is a statutory regulator that evaluates, inspects and regulates health and social care services in England.^10^, ^11^ Cases with an allocated CQC ID or a UPRN associated with a CQC ID were allocated as care home cases. Individuals with a BLPU from a private residential dwelling (at the time of testing) were extracted as controls.

### 2.3 Hospitalisation linkage

All COVID‐19 episodes were linked to the UKHSA's Hospital‐Onset COVID (HOCOVID) dataset, which pulls daily feeds on hospitalised individuals with a positive SARS‐CoV‐2 test from the Secondary Uses Service and the Emergency Care Data Set to obtain information on hospital admissions.^12^, ^13^, ^14^ Cases that linked to this dataset and had a positive SARS‐CoV‐2 test within 14 days or less before hospital admission were included in the study. The total number of hospitalisations was calculated for each individual and for each episode of COVID‐19.

### 2.4 Vaccination status

The data were linked to the National Immunisation Management System that UKHSA uses to record COVID‐19 vaccination status for all individuals with a National Health Service (NHS) number in England.^15^ Individuals that did not link to the dataset were marked as having an unknown vaccinations status. Vaccination status was calculated at the time of the onset of the earliest positive specimen date of the last episode.

### 2.5 Waves of infections

To account for the variants circulating at the time of infection, the following time periods were allocated as different waves of COVID‐19 variants throughout the study period and were calculated based on the earliest positive specimen date of the last episode^16^:

Wild type: 02 February 2020–30 July 2020.
Alpha: 31 July 2020–30 April 2021.
Delta: 01 May 2021–30 November 2021.
Omicron BA1: 01 December 2021–28 February 2022.
Omicron BA2: 01 March 2022–31 March 2022.

Furthermore, throughout the study period, access to testing varied. There was limited access to testing at the beginning of the pandemic when the wild variant was circulating.

### 2.6 Deaths associated with COVID‐19

Data for the collation of COVID‐19 death data are obtained from four sources: reporting from NHS hospitals via a dedicated COVID‐19 patient notification system; direct notification to UKHSA from local Health Protection Teams; NHS Demographic Batch Service tracing of COVID‐19 positive test data against the NHS Spine; ONS registered deaths with COVID‐19 mentioned on the death certificate. The COVID‐19 deaths dataset includes all deaths that occurred within 28 days of the earliest positive COVID‐19 test of the most recent COVID‐19 episode.^17^, ^18^ To calculate a crude case fatality rate (CFR), deaths linked by NHS number were divided by the total number of cases in both property settings. A total of 4673 of all 272,182 recorded deaths (1.17%) between 02 February 2020 and 28 April 2022 were reported where the specimen was unable to be linked to the line list and therefore were not included in the analyses.

### 2.7 Descriptive and statistical analyses

We first evaluated the total number of individuals who experience one or more SARS‐CoV‐2 infections while living in a care home for learning disabilities and or autism. Numbers of cases were observed over time, by age group and whether they were involved in an outbreak (two or more cases within 14 days of the previous case resident at the same UPRN).

Both residential care home cases and individuals in private residential dwellings who have tested positive for COVID‐19 were described by age, sex, ethnicity, region, hospitalisation status and vaccination status.

To evaluate the excess risk of death, univariable and multivariable logistic regression analysis, comparing the odds of death among of those living in residential care for service users with learning disabilities and/or autism who had tested positive for COVID‐19 with those with COVID‐19 cases living in a private residence, was evaluated.

Age, sex, ethnicity, IMD, region, number of COVID‐19 hospitalisations (for all episodes if there was a reinfection), vaccination status, month and year of the episode, variant wave and the maximum number of episodes, which could act as confounders, were considered for regression adjustment in the final multivariable model.

Three interaction parameters were assessed to identify the interaction between individuals with learning disabilities and living in a care home with the age group of COVID‐19 cases, with the variant wave and with the vaccination status. We calculated adjusted odds ratios using a post‐estimation analysis to estimate the odds of death among individuals with a learning disability and/or autism by age group and variant wave. We also conducted a sensitivity analysis evaluating the odds of death among unvaccinated during the period where wild type and alpha variants were dominant as there were a higher proportion of unvaccinated individuals during these periods.

## 3 RESULTS

### 3.1 Descriptive analyses

**FIGURE 1:**
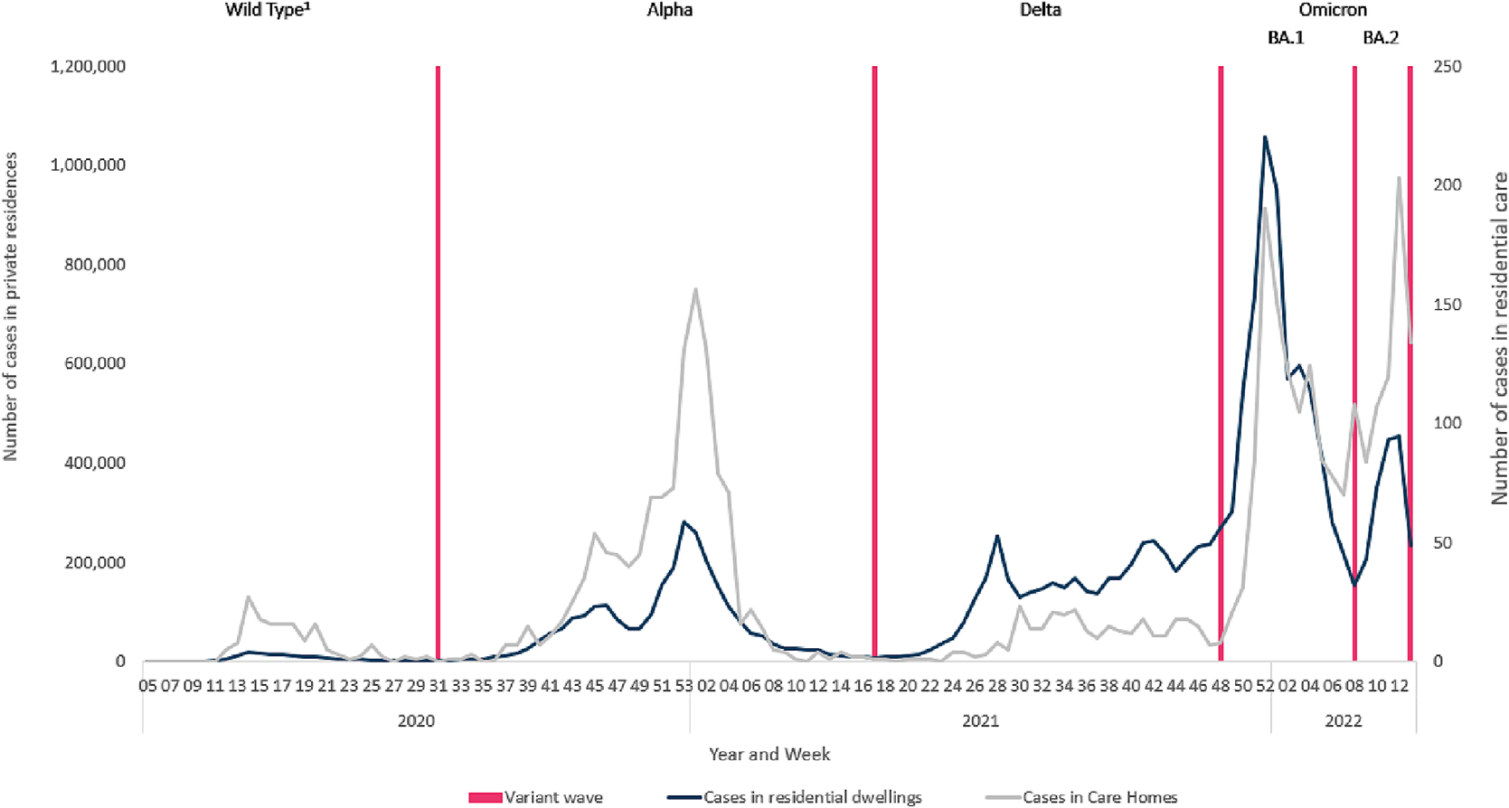
Total number of episodes of COVID‐19 among individuals living in private dwellings and living in care homes specialising in learning disabilities and/or autism from 02 February 2020 to 31 March 2022 in England. Testing was more limited during the wild type wave.

From 02 February 2020 to 30 March 2022, a total of 3501 (0.02%) individuals' resident in a care home specialising in learning disabilities and/or autism tested positive for COVID‐19. During the same period, 15,403,464 (99.98%) cases in individuals living in a private residential dwelling were identified. Figure 1 displays the trends in the number of cases across the pandemic waves, based on the earliest episode if they had a reinfection and shows similar trends in COVID‐19 cases over time.

Of the 3501 care home residents with learning disabilities and/or autism, 3317 individuals had a single infection of SARS‐CoV‐2, while 183 individuals had a reinfection and 1 individual had three episodes, totalling to 3686 infections of which 5.0% were reinfections (Table S1). A total of 5.0% of cases living in residential dwellings also had one or more reinfections. A total of 2113 (60.4%) cases were male, and 1378 were female. The age of cases ranged from less than 1 year old to 99 years old with the majority (77.8%) being adults between 30–69 years (Table 1); 69 cases were in children and adolescents less than 19 years old.

**TABLE 1:** Age groups of all individuals testing positive and deaths with COVID‐19 residing in a care home for learning disabilities and/or autism based on the age of the earliest positive specimen date of the earliest episode from 02 February 2020 to 31 March 2022 in England (*N* = 3501).

Age group (years)	No. of individuals with COVID‐19	%	No. of deaths with COVID‐19	% of deaths in all residential settings
<10	15	0.43	—	0.00
10–19	54	1.54	—	0.00
20–29	328	9.37	<10	0.38
30–39	471	13.45	—	0.00
40–49	602	17.2	10	0.48
50–59	990	28.28	24	0.41
60–69	661	18.88	26	0.21
70–79	301	8.6	22	0.09
80+	78	2.23	<10	0.02
Missing	1	0.03	—	0.00
Total	3501	100	90	0.10

A total of 90 individuals living in care homes for autism and/or learning disabilities died compared with 92,352 living in residential dwellings leading to a crude CFR of 2.57% and 0.6%, respectively (Table S1).

Of the 3686 infections occurring in care homes for learning disabilities and/or autism, 2962 (80.4%) were part of an outbreak where two or more cases occurred at the same residential address within a 14‐day period compared with 8,689,178 (53.7%) among individuals living in private dwellings (Table S2). The 2962 COVID‐19 episodes in care homes for learning disabilities and/or autism were part of 770 outbreaks at 632 care homes with the same UPRN and CQC ID, with an overall median size of three cases per outbreak lasting on average 4 days between the first and final infection (Table 2).

**TABLE 2:** Total number of outbreaks in care homes for individuals with learning disabilities and/or autism and the rage, median and interquartile rage of the size and duration of these outbreaks in England from 02 February 2020 to 31 March 2022.

COVID‐19 cases	Total number of outbreaks	Outbreak size (no. of cases)			Duration of outbreak (days)		
		Range	Median	IQR	Range	Median	IQR
Care home	770	1 to 33	3	2 to 5	1 to 38	4	0 to 7
Private dwelling^a^	3,389,006	1 to 10	2	2 to 3	0 to 134	3	1 to 6

^a^ Private dwellings were restricted to where there were 10 or less cases as some block apartment/flats fall under the same Unique Property Reference Number and appear as one residence type.

Most care home cases resided in a facility with 5–20 beds with 48% of cases and episodes occurring in care homes with 5–9 beds. The proportion of episodes involved in an outbreak increased with care home bed size (Table 3). A slightly higher proportion of female cases (82.1%) were part of a care home outbreak compared with males (79.9%).

**TABLE 3:** Total number of care home cases and episodes and those parts of an outbreak by care home size from 02 February 2020 to 31 March 2022 in England.

Number of beds in care home	Number of episodes	Number episodes part of an outbreak	Percentage of episodes within an outbreak	Number of cases	Number of cases in an outbreak	Percentage of episodes within an outbreak
<5	415	275	66.3	400	266	66.5
5 to <10	1786	1429	80.0	1710	1372	80.2
10 to <20	1008	818	81.2	947	776	81.9
20 to <30	252	233	92.5	233	215	92.3
30 to <40	129	119	92.2	119	110	92.4
40 to <50	96	88	91.7	92	86	93.5

### 3.2 Risk of death

The postestimation analysis found that the odds of death were 9.7 and 8.4 times greater among individuals <40 years old and 40–59 years old living in a care home for learning disabilities and/or autism compared with those living in a private setting in the same age groups, respectively (Table 4).

**TABLE 4:** Adjusted odds of death due to COVID‐19 among individuals with learning disabilities and/or autism living in a care home compared with those living in a private dwelling in England from 02 February 2020 to 31 March 2022.

Characteristics			Care home cases (%)	Private dwelling cases	Adjusted odds of death among individuals with learning disabilities
Age	<40		868 (24.8)	9,127,591 (59.3)	9.74 (1.33–71.52)
	40–59		1592 (45.5)	4,368,507(28.4)	8.41 (5.64–12.56)
	60+		1041 (29.7)	1,907,366 (12.4)	0.82 (0.58–1.17)
Wave	Wild type	(02.02.20–30.07.20)	171 (4.9)	186,397 (1.2)	1.81 (0.99–3.31)
	Alpha	(31.07.20–30.04.21)	1337 (38.2)	3,104,955 (20.2)	1.91 (1.32–2.78)
	Delta	(01.05.21–30.11.21)	321 (9.2)	4,572,061 (29.7)	1.50 (0.37–6.12)
	BA1	(01.12.21–28.02.22)	1111 (31.7)	6,051,476 (39.3)	0.32 (0.04–2.31)
	BA2	(01.03.22–31.03.22)	561 (16.0)	1,488,575 (9.7)	2.09 (0.73–5.96)
Vaccination status	Unknown/unlinked		454 (13.0)	1,001,739 (6.5)	1.65 (1.01–2.70)
	Unvaccinated		177 (5.1)	3,215,008 (20.9)	4.70 (2.74–8.06)
	Dose 1		1290 (36.9)	4,526,374 (29.4)	0.86 (0.35–2.07)
	Dose 2		369 (10.5)	3,692,559 (24.0)	1.22 (0.30–4.99)
	2 or 3 Doses + early booster		1211 (34.6)	2,967,784 (19.3)	1.59 (0.69–3.65)

Furthermore, the odds of death also varied throughout the course of the pandemic and with the variant type, where the odds of death were nearly two times greater among cases living in a care home for learning disabilities and/or autism during the alpha wave compared with those living in residential dwellings (Table 4). There was no significant increase in the odds of death among vaccinated cases residing in care home compared with their counterparts in private housing. However, unvaccinated individuals in a care home for learning disabilities and/or autism had an increased odds of death of 4.70 compared with those unvaccinated living in private dwellings. Results from the sensitivity analysis where we included cases between 02 February 2020 to 30 April 2021 (wild type and alpha waves) still showed an increased odds of death among unvaccinated residents of care homes for learning disabilities and/or autism of 3.90 (confidence interval [2.17, 7.00]) compared with those unvaccinated living in residential dwellings.

## 4 DISCUSSION

Throughout the pandemic, various control measures in care homes were set in place to reduce the transmission of COVID‐19, such as the use of personal protective equipment, routine testing, vaccinations and limited visiting arrangements, causing stress on residents and their families.

Our study identified over 3500 individuals diagnosed with SARS‐CoV‐2 while living in a care home for living disabilities and/or autism. A total of 80.4% of COVID‐19 episodes were part of an outbreak, considerably higher than for individuals living in their own home, illustrating the vulnerability from living in an institutional setting. Although this a likely reflection of the higher number residents in these facilities, it is also likely to reflect the nature of the care received, which would have exposed these individuals to many different staff, each with their own external exposures to COVID‐19.

Most care home cases were found in care homes with 5–20 beds, rather than in large care homes, though a greater proportion of cases living in larger care homes were part of an outbreak. It should be noted that the National Institute for Health Care Excellence guidance (CG142) states that residential care for autistic adults should be provided in smaller community‐based units with no more than six residents per unit, which may explain the greater number of cases in smaller care homes in this study.^19^ Further research is needed to evaluate SARS‐CoV‐2 infections among care home staff and the transmission between staff members and individuals with learning disabilities and/or autism care home residents, and vice‐versa.

In this study we evaluated the odds of death among individuals residing in a care home for people with learning disabilities and/or autism compared with those living in private dwellings as the recording of COVID‐19 deaths is a representative estimate among the two populations. However, although there was free access to community testing throughout the study period, care homes routinely tested both residents and staff members and therefore could have detected more cases, particularly cases with mild infection or asymptomatic, factors which could have reduced the estimated CFR.

By observing different interactions, we found that individuals residing in a care home for learning disabilities and/or autism had a disproportionally increased risk of death, particularly among those 40 years old and younger where the odds of death was close to 10 times higher than age‐matched counterparts living in their own home. This concurs with previous research that used different methods for identifying cases with learning disabilities.^7^, ^20^

Upon reviewing the interaction of variant waves on the risk of death, a significant difference was only observed during the alpha wave where the risk of death was nearly twice as high among those residing in a care home specialising in learning disabilities and/or autism versus those living in private dwellings. The first two waves of the COVID‐19 pandemic had a substantial impact on elderly people living in care homes specialising in disabilities and/or autism where high numbers of death occurred, most likely due to the limited knowledge on COVID‐19 at the time, high transmission through frequent close contact, no vaccination available and shortages in masks and personal protective equipment for carers. Changes in adult social care guidance, the introduction of COVID‐19 vaccines and the varying disease severity with each variant may have resulted in the insignificant odds of death between those living in care homes for learning disabilities and/or autism and individuals in residential dwellings in following waves of infection.^21^, ^22^

Furthermore, individuals living in care homes for learning disabilities and/or autism that were unvaccinated or with an unknown vaccination status had four times the odds of death compared with those unvaccinated or with unknown vaccination status living in residential dwellings. This was also observed during the wild type and alpha variant waves when vaccinations were either not available or the first vaccinations were being rolled out in England. The odds of death among vaccinated individuals were not significantly different for those with learning disabilities and/or autism in care homes versus their counterparts residing in residential settings, thus indicating that the vaccination programme may have helped reduce inequities in the burden of disease.

To evaluate cases living in care homes specialising in learning disabilities and/or autism, we used the CQC register of regulated facilities. It is possible that some care homes have been missed due to information on the CQC website being out of date due to changing care facilities. It was necessary to only include care homes with the single service users of individuals with learning disabilities and/or autism; therefore, this study cannot draw conclusions for those living in care homes serving multiple service user types. Furthermore, in these analyses we only analyse individuals that had a single infection or where the reinfection was at the same property type. Any cases that may have lived in both a care home and private residence throughout the study period would not have been included; however, the impact of this is likely to be minimal due to the small number of cases living in these two settings, which accounts for approximately 5% of care home infections.

Our study does have some further limitations. Deaths reported by ONS or NHS England without a test result or with a test result that did not match to the line list of COVID19 patients were unable to be linked to receive information on their type of residence and were therefore not included in the analyses. It is likely that these individuals are more likely to have resided in residential dwellings due to increased testing in care home facilities, which may overestimate the odds of death. That being said, a CQC report indicated a total of 195 deaths COVID‐19 deaths between 2019 and 2020 among individuals with learning disabilities of whom some may be autistic and where learning disability was indicated on the death notification form.^23^ It is likely that these estimates include individuals from multiple service care homes and possibly the stricter 28‐day death definition could have underestimated the odds of death in our analyses. Furthermore, it has been noted that under the provisions of the 2014 Care Act, individuals who purchase care from non‐registered providers are not included in the reported CQC data and therefore could further underestimate the risk of death.^20^ Further analyses using contact tracing information and information on the total number of residents residing in each care home could help to understand transmission patterns in these settings to best guide policies in this area. It was also not possible to detect the mode of transmission of disease between carers and residents in care home settings where residents with learning disabilities and/or autism were infected.

Due to the nature of the data, we were unable to identify learning disabilities and/or autism at the individual case level; therefore, we could not compare individuals with such disabilities living in private homes and institutional settings. Further linkage to datasets, such as GP records would be useful to further identify individuals and compare the risk of COVID‐19 among individuals across various property types. Finally, further information on the severity of learning disability of the cases to stratify risk within these residences would be of value.

Previous studies show that there remains a significant gap on where people with learning disabilities reside in any health records. Evidence generation of the impact of COVID‐19 on those with learning disabilities and/or autism in England, by initiatives such as the Learning Disabilities Mortality Review and OpenSAFELY, have emphasised that people living with learning disabilities and/or autism experience risk differently based on several factors, including type of residence.^7^, ^24^ These analyses used different data sources for the approximation for the type of residence as the learning disabilities were not systematically recorded. Our analysis adds to the evidence base by studying those infected by COVID‐19 while living in residential care homes, utilising information from large national surveillance systems, thus adding to the triangulation of data used to evaluate the impact of COVID‐19 on learning disabilities.

Our study adds value to understanding the necessity of rapid infection control in specialised care homes in order to reduce COVID‐19 infection and mortality. The novel approach of linking address information from cases to obtain information on property types could in future help identify cases and clusters of COVID‐19 and any other disease for more rapid public health evaluation. Our study indicates that individuals residing in a care home for learning disabilities and/or autism have experienced greater risk of death from COVID‐19 compared with individuals living in private residences. However, risk is not uniform and factors influencing risk must be considered when designing accessible public health guidance, vaccination and healthcare to better protect this population from COVID‐19.

## AUTHOR CONTRIBUTIONS

**Elise Tessier:** Conceptualization; data curation; formal analysis; investigation; methodology; project administration; resources; software; validation; visualization; writing–original draft; writing–review and editing. **Harriet Webster:** Conceptualization; data curation; formal analysis; investigation; methodology; project administration; resources; software; validation; visualization; writing–original draft; writing–review and editing. **Nurin Abdul Aziz:** Conceptualization; investigation; methodology; formal analysis; validation; visualization; writing–original draft; writing–review and editing. **Joe Flannagan:** Conceptualization; investigation; methodology; project administration; resources; writing–review and editing. **Asad Zaidi:** Conceptualization; data curation; formal analysis; software; visualization; writing–review and editing. **Andre Charlett:** Formal analysis; investigation; methodology; software; validation; visualization; writing–review and editing. **Gavin Dabrera:** Conceptualization; investigation; methodology; project administration; supervision; writing–original draft; writing–review and editing. **Theresa Lamagni:** Conceptualization; project administration; supervision; writing–original draft; writing–review and editing.

## CONFLICT OF INTEREST STATEMENT

We declare no conflicts of interest.

### PEER REVIEW

The peer review history for this article is available at https://www.webofscience.com/api/gateway/wos/peer-review/10.1111/irv.13139.

## Supporting information


**Table S1.** The total number of care home cases and controls testing positive for SARS‐CoV‐2 by age, sex, ethnicity, region, number of beds within a care home, hospitalisation status, death by 28 days, and vaccination status in England from 02 February 2020 to 31 March 2022.

**Table S2.** Total number of care home cases and episodes that were part of an outbreak in England from 02 February 2020 to 31 March 2022.

## Data Availability

The data that support these studies that were collected as part of a public health response are considered sensitive and not made publicly available. Reasonable requests for access to anonymised data and data dictionary will be considered by the authors to allow all results to be reproduced.
