# Supplementary material for: The impact of COVID‐19 on residents of long‐term care facilities with learning disabilities and/or autism
Source: Influenza Other Respir Viruses. 2023 Apr 26;17(4):e13139. doi: 10.1111/irv.13139 (PMC10133727; doi:10.1111/irv.13139)
Supplement: Supplementary file 1 — Table S1. The total number of care home cases and controls testing positive for SARS‐CoV‐2 by age, sex, ethnicity, region, number of beds within a care home, hospitalisation status, death by 28 days, and vaccination status in England from 02 February 2020 to 31 March 2022. [file IRV-17-e13139-s002.docx]

**Supplementary Table 1.** The total number of care home cases and controls testing positive for SARS-CoV-2 by age, sex, ethnicity, region, number of beds within a care home, hospitalisation status, death by 28 days, and vaccination status in England from 02 February 2020 to 31 March 2022.

| **Characteristic** |  | **Care home cases** | **Percent of all Cases (%)** | | **Residential cases** | **Percent of all cases (%)** | **Total** |
| --- | --- | --- | --- | --- | --- | --- | --- |
| Age group | <40 | 868 | 24.79 | 9,127,591 | | 59.26 | 9,128,459 |
|  | 40-59 | 1,592 | 45.47 | 4,368,507 | | 28.36 | 4,370,099 |
|  | 60+ | 1,041 | 29.73 | 1,907,366 | | 12.38 | 1,908,407 |
| Sex | Female | 1,378 | 39.36 | 8,244,399 | | 53.52 | 8,245,777 |
|  | Male | 2,113 | 60.35 | 7,116,568 | | 46.20 | 7,118,681 |
|  | Unknown | 10 | 0.29 | 42,497 | | 0.28 | 42,507 |
| Ethnicity | White | 2,847 | 81.32 | 12,244,466 | | 79.49 | 12,247,313 |
|  | Asian/ Asian British | 96 | 2.74 | 1,347,908 | | 8.75 | 1,348,004 |
|  | Black / African / Caribbean | 124 | 3.54 | 494,588 | | 3.21 | 494,712 |
|  | Mixed/ Multiple ethnicity | 36 | 1.03 | 390,856 | | 2.54 | 390,892 |
|  | Other | 16 | 0.46 | 191,050 | | 1.24 | 191,066 |
|  | Unknown | 382 | 10.91 | 734,596 | | 4.77 | 734,978 |
| IMD | 1 | 241 | 6.88 | 1,517,333 | | 9.85 | 1,517,574 |
|  | 2 | 354 | 10.11 | 1,551,816 | | 10.07 | 1,552,170 |
|  | 3 | 405 | 11.57 | 1,564,750 | | 10.16 | 1,565,155 |
|  | 4 | 413 | 11.80 | 1,536,751 | | 9.98 | 1,537,164 |
|  | 5 | 519 | 14.82 | 1,525,152 | | 9.90 | 1,525,671 |
|  | 6 | 475 | 13.57 | 1,539,295 | | 9.99 | 1,539,770 |
|  | 7 | 323 | 9.23 | 1,515,190 | | 9.84 | 1,515,513 |
|  | 8 | 357 | 10.20 | 1,543,459 | | 10.02 | 1,543,816 |
|  | 9 | 258 | 7.37 | 1,552,019 | | 10.08 | 1,552,277 |
|  | 10 | 154 | 4.40 | 1,554,651 | | 10.09 | 1,554,805 |
|  | Unknown | 2 | 0.06 | 3,048 | | 0.02 | 3,050 |
| Latest Episode | 1 | 3,317 | 94.74 | 14,632,124 | | 94.99 | 14,635,441 |
|  | 2 | 183 | 5.23 | 763,281 | | 4.96 | 763,464 |
|  | 3 | 1 | 0.03 | 7,986 | | 0.05 | 7,987 |
|  | 4 | 0 | 0.00 | 73 | | 0.00 | 73 |
| Region | London | 434 | 12.40 | 2,319,980 | | 15.06 | 2,320,414 |
|  | East Midlands | 285 | 8.14 | 1,330,825 | | 8.64 | 1,331,110 |
|  | East of England | 382 | 10.91 | 1,816,901 | | 11.80 | 1,817,283 |
|  | North East | 153 | 4.37 | 782,940 | | 5.08 | 783,093 |
|  | North West | 280 | 8.00 | 2,132,173 | | 13.84 | 2,132,453 |
|  | South East | 814 | 23.25 | 2,436,975 | | 15.82 | 2,437,789 |
|  | South West | 392 | 11.20 | 1,431,534 | | 9.29 | 1,431,926 |
|  | West Midlands | 307 | 8.77 | 1,597,540 | | 10.37 | 1,597,847 |
|  | Yorkshire and Humber | 454 | 12.97 | 1,515,608 | | 9.84 | 1,516,062 |
|  | Unknown | 0 | 0.00 | 38,988 | | 0.25 | 38,988 |
| Hospitalisation | None | 3,456 | 98.71 | 15,333,930 | | 99.55 | 15,337,386 |
|  | 1 hospitalisation | 40 | 1.14 | 65,762 | | 0.43 | 65,802 |
|  | 2 or more | 5 | 0.14 | 3,772 | | 0.02 | 3,777 |
| 28-day death | Dead | 90 | 2.57 | 92,352 | | 0.60 | 92,443 |
|  | Alive | 3,410 | 97.40 | 15,311,112 | | 99.40 | 15,314,522 |
| Vaccination status | Unknown/ Unlinked | 454 | 12.97 | 997,874 | | 6.48 | 998,328 |
|  | Unvaccinated | 177 | 5.06 | 3,215,882 | | 20.88 | 3,216,059 |
|  | Dose 1 | 1,163 | 33.22 | 4,161,588 | | 27.02 | 4,162,751 |
|  | Dose 2 | 373 | 10.65 | 3,832,479 | | 24.88 | 3,832,852 |
|  | 2 or 3 Doses + early booster | 1,334 | 38.10 | 3,195,641 | | 20.75 | 3,196,975 |
| Variant wave | Wild type | 171 | 4.88 | 186,397 | | 1.21 | 186,568 |
|  | Alpha | 1337 | 38.19 | 3,104,955 | | 20.16 | 3,106,292 |
|  | Delta | 321 | 9.17 | 4,572,061 | | 29.68 | 4,572,382 |
|  | Omicron BA1 | 1,111 | 31.73 | 6,051,476 | | 39.29 | 6,052,587 |
|  | Omicron BA2 | 561 | 16.02 | 1,488,575 | | 9.66 | 1,489,136 |
