# Supplementary material for: The impact of COVID‐19 on residents of long‐term care facilities with learning disabilities and/or autism
Source: Influenza Other Respir Viruses. 2023 Apr 26;17(4):e13139. doi: 10.1111/irv.13139 (PMC10133727; doi:10.1111/irv.13139)
Supplement: Supplementary file 2 — Table S2. Total number of care home cases and episodes that were part of an outbreak in England from 02 February 2020 to 31 March 2022. [file IRV-17-e13139-s001.docx]

**Supplementary Table 2.** Total number of care home cases and episodes that were part of an outbreak in England from 02 February 2020 to 31 March 2022.

| **Episode number** | **Total no. episodes** | **No. episodes part of an outbreak** | **%** | **Total no. cases based on the latest episode** | **Total number of cases with a reinfection** | **%** |
| --- | --- | --- | --- | --- | --- | --- |
| 1 | 3,501 | 2,825 | 80.7 | 3,317 | 2,694 | 81.2 |
| 2 | 184 | 136 | 74.3 | 183 | 130 | 71.0 |
| 3 | 1 | 1 | 100.0 | 1 | 1 | 100.0 |
| Total | 3,686 | 2,962 | 80.4 | 3,501 | 2,825 | 80.7 |
